# Construction of a Recombinant Japanese Encephalitis Virus with a Hemagglutinin-Tagged NS2A: A Model for an Analysis of Biological Characteristics and Functions of NS2A during Viral Infection

**DOI:** 10.3390/v14040706

**Published:** 2022-03-29

**Authors:** Xiaochun Ma, Chenxi Li, Qiqi Xia, Yan Zhang, Yang Yang, Abdul Wahaab, Ke Liu, Zongjie Li, Beibei Li, Yafeng Qiu, Jianchao Wei, Zhiyong Ma

**Affiliations:** Shanghai Veterinary Research Institute, Chinese Academy of Agricultural Sciences, Shanghai 200241, China; maxiaochun126@126.com (X.M.); lichenxihsy@outlook.com (C.L.); xiaqiqi1996@163.com (Q.X.); 18672550235@163.com (Y.Z.); 15776623916@163.com (Y.Y.); abdul.wahaab@uaf.edu.pk (A.W.); liuke@shvri.ac.cn (K.L.); lizongjie@shvri.ac.cn (Z.L.); lbb@shvri.ac.cn (B.L.); yafengq@shvri.ac.cn (Y.Q.)

**Keywords:** Japanese encephalitis virus, nonstructural protein 2A (NS2A), HA-tag, recombinant virus, NS1’, replication, model

## Abstract

Nonstructural protein 2A (NS2A) of the Japanese encephalitis virus (JEV) contributes to viral replication and pathogenesis; however, a lack of NS2A-specific antibodies restricts studies on the underlying mechanisms. In this study, we constructed a recombinant JEV with a hemagglutinin (HA)-tagged NS2A (JEV-HA/NS2A/∆NS1’) to overcome this challenge. An HA-tag was fused to the N-terminus of NS2A (HA-NS2A) at the intergenic junction between NS1 and NS2A. A peptide linker, “FNG”, was added to the N-terminus of HA-tag to ensure correct cleavage between the C-terminus of NS1 and the N-terminus of HA-NS2A. To avoid the side effects of an unwanted NS1’ tagged with HA (HA-NS1’), an alanine-to-proline (A30P) substitution was introduced at residue 30 of NS2A to abolish HA-NS1’ production. The HA-tag insertion and A30P substitution were stably present in JEV-HA/NS2A/∆NS1’ after six passages and did not exhibit any significant effects on viral replication and plaque morphology. Taking advantage of HA-NS2A, we examined the activities of NS2A during JEV infection in vitro using anti-HA antibodies. NS2A was observed to be localized to the endoplasmic reticulum and interact with viral NS2B and NS3 during virus infection. These data suggest that JEV-HA/NS2A/∆NS1’ can serve as a model for the analysis of the biological characteristics and functions of NS2A in vitro during JEV infection.

## 1. Introduction

Japanese encephalitis virus (JEV) is a mosquito-borne zoonotic virus that is most prevalent in Southeast Asia and the Western Pacific [1,2]. JEV invades the central nervous system and causes encephalitis in humans and reproductive disorders in pigs. In human infections, approximately 30% cases are fatal, especially in children [3]. JEV is one member of the genus *Flavivirus* of the family *Flaviviridae* that comprises more than 70 species, including Zika virus (ZIKV), dengue virus (DENV), and yellow fever virus (YFV) [4]. The JEV genome is a single-stranded, positive sense RNA that encodes a single polyprotein. Following translation, the polyprotein is proteolytically cleaved by host and/or viral proteases at various junction sites between each viral protein into three structural proteins (C, prM/M, and E) and seven nonstructural proteins (NS1, NS2A, NS2B, NS3, NS4A, NS4B, and NS5) [5].

Flavivirus NS2A is a hydrophobic protein approximately 22 kDa in size, which contains five integral transmembrane segments across the lipid bilayer of the endoplasmic reticulum (ER) membrane [6]. Moreover, flavivirus NS2A is involved in the recruitment of viral RNA, structural proteins, and NS2B-NS3 protease to the site of virion assembly and the coordination of nucleocapsid and virus formation [7,8], playing an essential role in viral RNA synthesis and virion assembly [6,7,8,9,10,11,12,13]. In addition, NS2A participates in the evasion of innate immunity and disease pathogenesis [8]. The NS2A of ZIKV and DENV inhibits the induction of type-I interferon [14,15]. Moreover, ZIKV NS2A impairs mammalian cortical neurogenesis through depleting adherens junction proteins [16]. A conserved slippery heptanucleotide motif is present at the beginning of flavivirus NS2A gene, and is responsible for the production of a derivative NS1 (NS1’) [17]. Mutations in the GCC codon for alanine at amino acid position 30 of NS2A by codon CCA disrupts the frameshift-stimulating pseudoknot structure, thereby abolishing the synthesis of NS1’ [18]. 

Previous studies have demonstrated that JEV NS2A is involved in viral growth and tissue tropism in vitro [19]. A single silent mutation, G66A, in the NS2A gene was found to abolish NS1’ production in vitro and reduce neurovirulence and neuroinvasiveness in mice [20]. NS2A also specifically inhibits the double-stranded RNA-activated protein kinase, PKR to mediate the host antiviral response [21]. These observations suggest the essential role of NS2A in JEV infection; however, the precise role and underlying mechanism of NS2A in JEV infection remains largely unknown. The current knowledge of JEV NS2A is primarily extrapolated from other flaviviruses. One major challenge for the exploration of JEV NS2A functionality is a lack of specific antibodies. As an alternative solution, NS2A has been labeled with different tags (e.g., Flag [22] and hemagglutinin [HA] [21]) and is ectopically expressed in vitro to analyze its functions. However, ectopically expressed viral proteins may not completely reflect the functions and biological activities of NS2A during viral infection. During the process of viral infection, viral proteins can interact with a variety of other molecules, including viral and host proteins required for replication. For example, the ectopic expression of ZIKV NS3 exhibits mitochondrial localization that is quite different from its ER localization in ZIKV-infected cells [23]. 

Since flavivirus NS2A is a membrane protein localized to the ER, the generation of specific antibodies remains a challenge [8]. We also failed to produce antibodies against JEV NS2A despite attempting to use different approaches. In the present study, we constructed a recombinant JEV with HA-tagged NS2A using an infectious cDNA clone of the SD12 strain to overcome the lack of specific antibodies against JEV NS2A.

## 2. Materials and Methods

### 2.1. Cells, Virus, and Antibodies

Baby hamster kidney (BHK-21) cells were cultured in Dulbecco’s modified Eagle’s medium (DMEM) containing 10% fetal bovine serum (FBS) at 37 °C with 5% CO_2_. The JEV SD12 strain was grown and titrated using a 50% tissue culture infective dose (TCID_50_) assay in BHK-21 cells. The commercial antibodies used in this study consisted of: mouse anti-HA monoclonal antibody (H9658, Sigma, St. Louis, MO, USA); rabbit anti-calnexin polyclonal antibody (C4731, Sigma); rabbit anti-Erlin-2 antibody (EPR8088, Abcam, Shanghai, China); mouse anti-β-actin monoclonal antibody (Proteintech Group, Chicago, IL, USA); rabbit anti-JEV C polyclonal antibody (GTX131368, GeneTex, St. Anthony, TX, USA); rabbit anti-JEV E polyclonal antibody (GTX125867, GeneTex); rabbit anti-JEV NS1 polyclonal antibody (GTX131370, GeneTex); mouse anti-JEV NS1 monoclonal antibody (GTX633820, GeneTex); rabbit anti-JEV NS2B polyclonal antibody (GTX125972, GeneTex); rabbit anti-JEV NS4A polyclonal antibody (GTX132028, GeneTex); and rabbit anti-JEV NS4B polyclonal antibody (GTX125865, GeneTex). Mouse anti-JEV prM polyclonal, mouse anti-JEV NS3 polyclonal, and rabbit anti-JEV NS5 polyclonal antibodies were prepared and preserved in our laboratory.

### 2.2. Construction of Recombinant JEV

The construction of recombinant JEV with HA-tagged NS2A was performed as previously described [8]. Briefly, four pairs of primers targeting the highly conserved sequences of the JEV SD12 stain (GenBank No. MH753127) (Supplementary Table S1) were used to amplify four overlapping fragments covering the full-length gene of the strain, respectively. The fragments amplified by reverse transcription-polymerase chain reaction (RT-PCR) were inserted into a pOK12 vector to generate four recombinant plasmids, which were pJEV-I (nucleotides 1–2361 with the addition of a T7 promoter at the 5′ terminus), pJEV-II (nucleotides 2332–4497), pJEV-III (nucleotides 4468–7664), and pJEV-IV (nucleotides 7636–10,965) (Supplementary Figure S1A). A full-length infectious cDNA clone of the SD12 strain was generated in vitro by splicing four fragments through the homologous recombination with a Gibson Assembly Cloning Kit (New England Biolabs, Ipswich, MA, USA). Full-length viral RNA was transcribed in vitro from a full-length viral cDNA template with an mMessage mMachine T7 kit (Invitrogen, Carlsbad, CA, USA). BHK-21 cells were transfected with the resulting viral RNA transcripts using DMRIE-C (Invitrogen) to rescue the recombinant virus. To generate recombinant JEV with HA-tagged NS2A, two overlapping fragments (nucleotides 2332–3564, and 3550–4542) were amplified by PCR with a primer pair containing the HA-tag and linker sequences (Supplementary Table S1) using pJEV-II as a template and spliced through homologous recombination to generate the recombinant plasmid, pJEV-II-HA. pJEV-II-HA together with pJEV-I, pJEV-III, and pJEV-IV were used to generate recombinant virus, JEV-HA/NS2A (Supplementary Figure S1B). To generate recombinant JEV with HA-tagged NS2A and A30P substitution, two overlapping fragments (nucleotides 2332–3685, and 3667–4542) were amplified by PCR with a primer pair containing the sequences of A30P substitution (Supplementary Table S1) using pJEV-II-HA as a template and spliced through homologous recombination to generate the recombinant plasmid, pJEV-II-HA-A30P. pJEV-II-HA-A30P together with pJEV-I, pJEV-III, and pJEV-IV were used to generate recombinant virus JEV-HA/NS2A/∆NS1’ (Supplementary Figure S1C). All recombinant viruses rescued from BHK-21 cells were serially passaged six times, including three rounds of plaque-purification in BHK-21 cells and subsequently confirmed by Sanger sequencing (Invitrogen, Shanghai, China). Recombinant virus clones containing any unwanted mutations were discarded.

### 2.3. Growth Kinetics and Viral Plaque Assays

BHK-21 cells were infected with JEV at a multiplicity of infection (MOI) of 0.01 and cultured in DMEM containing 1% FBS. Supernatants were collected at different time points and titrated with TCID_50_ assays in BHK-21 cells. Viral plaque assays were performed as previously described [24].

### 2.4. Immunofluorescence and Western Blot Assays

BHK-21 cells were infected with JEV at an MOI of 0.01 and incubated for 24 h. The cells were washed three times with pre-chilled phosphate buffered saline and fixed in 4% paraformaldehyde for an immunofluorescence assay (IFA). The infected cells were harvested at the indicated time points and subjected to western blot analysis. IFA and western blots were performed as previously described [25]. 

### 2.5. Co-Immunoprecipitation

BHK-21 cells were infected with recombinant JEV and harvested at 48 h post-infection (hpi) for the co-immunoprecipitation assays. Briefly, the harvested cells were lysed in ice-cold RIPA buffer (P0013D, Beyotime, Shanghai, China) containing 1% NP40, 0.25% deoxycholate, and EDTA-free protease inhibitor cocktail (S8830, Sigma) and sonicated for 15 s on ice. Following a 30 min incubation on ice, the supernatants were collected by centrifugation at 12,000 rpm for 10 min at 4 °C and incubated with anti-HA antibodies pre-treated with Dynabeads Protein G (10004D, Invitrogen) in the presence or absence of RNase A (50 μg/mL) at 4 °C overnight. The presence of JEV NS1 RNA in the supernatant was examined by RT-PCR. The bead-bound immune complexes were collected using a DynaMag-2 magnet (12321D, Invitrogen), followed by washing five times with RIPA buffer, and subjected to western blotting. 

### 2.6. Detergent-Resistant Membrane Isolation

Detergent-resistant membrane isolation of JEV-infected cells was performed as described previously [26]. Briefly, BHK-21 cells were infected with JEV and harvested at 48 hpi for the isolation of detergent-resistant membranes (DRM). The harvested cells were lysed in TNE buffer (25 mM Tris-HCl [pH 7.5], 150 mM NaCl, and 5 mM EDTA) containing 0.5% Triton X-100 on ice for 30 min and subsequently homogenized with a Dounce homogenizer. The crude lysates were centrifuged at 1000× *g* at 4 °C for 10 min and the supernatants were mixed with 80% sucrose/TNE (wt/vol) to prepare the lysate-sucrose mixture containing a final concentration of 60% sucrose. A discontinuous sucrose gradient containing 80% sucrose/TNE, the lysate-sucrose mixture, 50%, 38%, and 10% sucrose/TNE was gently layered and subsequently ultracentrifuged at 100,000× *g* at 4 °C for 16 h in an SW41 rotor (Beckman Coulter, Carlsbad, CA, USA). The top five fractions that were considered to contain the DRM were collected and subjected to western blot analysis. The DRM was identified by antibodies specific to the ER lipid raft-associated protein 2 (Erlin-2).

### 2.7. Statistical Analysis

All data were processed using Graph Pad Prism 7.0 (GraphPad, La Jolla, CA, USA). A Student’s *t*-test was used for statistical analyses and a *p*-value < 0.05 was considered to be statistically significant.

## 3. Results

### 3.1. Construction of Recombinant JEV with HA-Tagged NS2A

**Figure 1:**
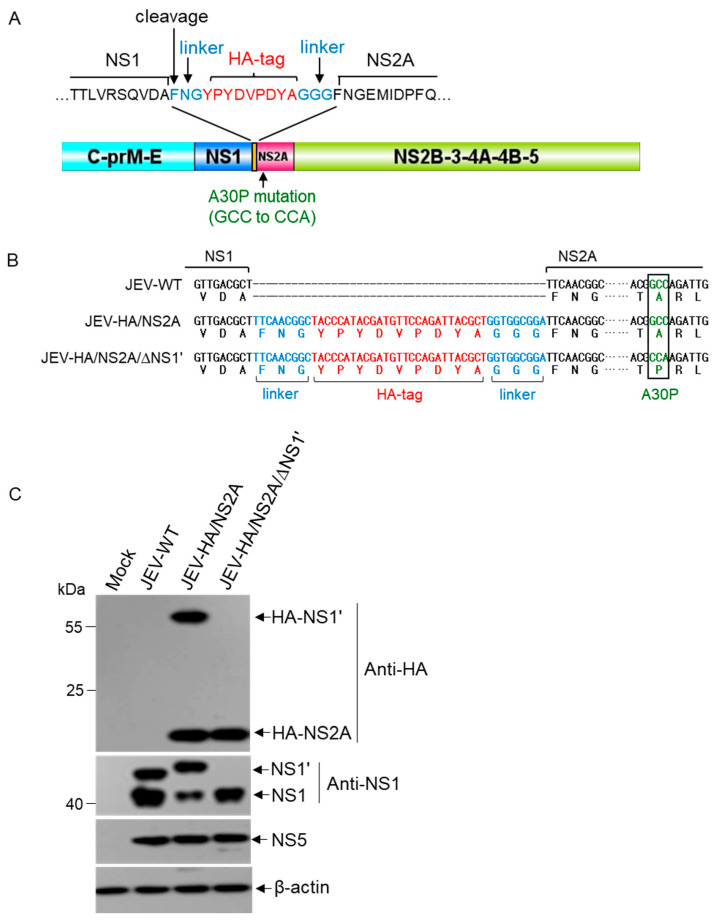
Construction of recombinant JEV with HA-tagged NS2A. (**A**) Schematic diagram of recombinant JEV with HA-tagged NS2A. (**B**) HA-tag, linkers, and A30P mutation sequences. (**C**) Detection of HA-NS2A expression by western blotting. BHK-21 cells were infected with the recombinant JEV and harvested for analysis of viral protein expression by western blotting.

To construct recombinant JEV with HA-tagged NS2A (JEV-HA/NS2A), an HA-tag was inserted into the intergenic junction between NS1 and NS2A. A peptide linker with the amino acid sequence, “FNG”, from the N-terminus of NS2A was fused to the N-terminus of HA-tag to ensure the correct cleavage between the C-terminus of NS1 and the N-terminus of HA-NS2A. A peptide linker of “GGG” was added to the C-terminus of the HA-tag to increase flexibility between the HA-tag and NS2A (Figure 1A,B). A full-length cDNA clone of JEV-HA/NS2A was generated by homologous recombination (Supplementary Figure S1) and was transcribed into RNA in vitro. Recombinant JEV-HA/NS2A was rescued from BHK-21 cells transfected with the resulting viral RNA transcripts. HA-NS2A expression in the cells infected with JEV-HA/NS2A was examined by western blots. A 17 kDa band corresponding to HA-NS2A was detected by anti-HA antibodies in the cells infected with JEV-HA/NS2A, but not in those infected with its parental strain (JEV-WT) (Figure 1C), suggesting that NS2A was labeled with an HA-tag. However, a 57 kDa band was also detected by anti-HA antibodies in cells infected with JEV-HA/NS2A (Figure 1C). 

It has been established that an RNA pseudoknot-mediated ribosomal frameshift event occurring between the codons 8 and 9 of NS2A gene results in the synthesis of a derivative NS1 (NS1’) protein, in which a 52 amino acid peptide is added to the C-terminus of NS1 [17,18]. Moreover, the insertion of an HA-tag between NS1 and NS2A might generate a derivative NS1’ tagged with HA (HA-NS1’). To test this hypothesis, NS1’ expression in the cells infected with JEV-WT and JEV-HA/NS2A was examined by western blotting with antibodies capable of detecting both NS1 and NS1’. As shown in Figure 1C, both NS1 and NS1’ were detected in cells infected with JEV-WT. The molecular weight of NS1 in the cells infected with JEV-HA/NS2A was identical to that in the cells infected with JEV-WT, suggesting the correct cleavage between NS1 and HA-NS2A. Meanwhile, a 57 kDa band that was slightly higher than NS1’ in molecular weight and corresponding to HA-NS1’ was present in the cells infected with JEV-HA/NS2A. These results indicate that the derivative HA-NS1’ was produced by JEV-HA/NS2A due to an HA-tag insertion.

The presence of HA-NS1’ might consequently interfere with the results when using anti-HA antibodies to analyze HA-NS2A functions. To eliminate HA-NS1’ production, a codon GCC for alanine at amino acid position 30 of NS2A was replaced with codon CCA for proline to generate an alanine 30-to-proline (A30P) substitution mutant of HA-NS2A (JEV-HA/NS2A/∆NS1’) (Figure 1B and Supplementary Figure S1C), in which the frameshift-stimulating pseudoknot structure was disrupted, abolishing HA-NS1’ synthesis [18]. HA-NS1’ expression in the cells infected JEV-HA/NS2A/∆NS1’ was examined by western blotting with both anti-HA and anti-NS1 antibodies. No band corresponding to HA-NS1’ was detected, compared with the cells infected with JEV-HA/NS2A (Figure 1C), suggesting that HA-NS1’ production was abolished. Overall, these data indicate that recombinant JEV-HA/NS2A/∆NS1’ harboring HA-NS2A and silenced in HA-NS1’ production was constructed.

### 3.2. Analysis of JEV-HA/NS2A/∆NS1’ Stability

**Figure 2:**
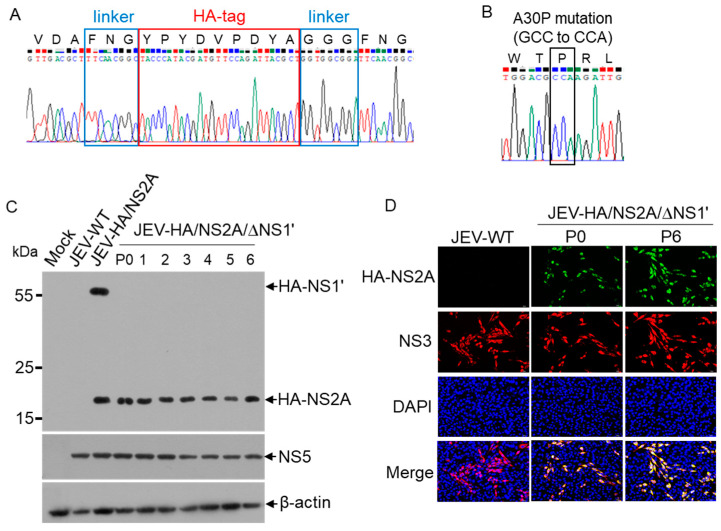
Analysis of stability of JEV-HA/NS2A/∆NS1’. The full-length gene sequence of JEV-HA/NS2A/∆NS1’ was amplified by RT-PCR from passage 6 and subjected to DNA sequencing. (**A**) Sequencing chromatogram of HA-tag insertion. (**B**) Sequencing chromatogram of A30P mutation. (**C**,**D**) BHK-21 cells were infected with the indicated recombinant JEV and subjected to western blots (**C**) and IFA analysis (**D**). Detection of HA-NS2A expression by IFA. HA-NS2A (green) and NS3 (red) were detected with anti-HA and anti-NS3 antibodies, respectively. The nuclei (blue) were stained with 4’,6-diamidino-2-phenylindole (DAPI).

JEV-HA/NS2A/∆NS1’ was serially passaged six times, including three rounds of plaque-purification on BHK-21 cells to examine its stability. The full-length gene sequence of JEV-HA/NS2A/∆NS1’ was amplified from the passage 6 cells and sequenced. The HA-tag insertion (Figure 2A) and A30P substitution (GCC to CCA) (Figure 2B) were observed to be present in passage 6 of JEV-HA/NS2A/∆NS1’, which suggests that JEV-HA/NS2A/∆NS1’ stably harbored mutations of interest. The cells infected with JEV-HA/NS2A/∆NS1’ were collected from each passage for an analysis of HA-NS2A expression by western blotting with anti-HA antibodies. HA-NS2A, but not HA-NS1’, was detected in each passage from passage 0 (p0) to passage 6 (p6) (Figure 2C). In addition, the expression of HA-NS2A was further examined by IFA with anti-HA antibodies. Fluorescent signals of HA-NS2A were detectable in cells infected with JEV-HA/NS2A/∆NS1’, but not with JEV-WT (Figure 2D). Overall, these data indicate that JEV-HA/NS2A/∆NS1’ stably harbored and expressed HA-NS2A.

### 3.3. Effects of HA-Tag Insertion and NS1’ Deletion on JEV Replication

**Figure 3:**
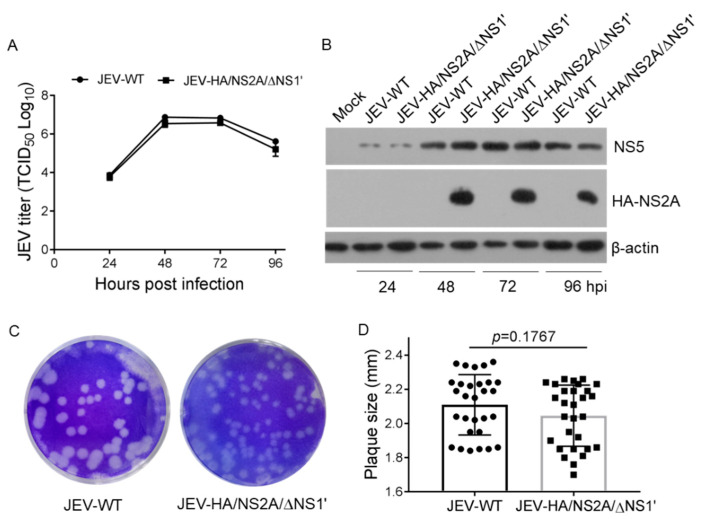
Replication of JEV-HA/NS2A/∆NS1’ in BHK-21 cells. (**A**,**B**) BHK-21 cells were infected with the indicated viruses at an MOI of 0.01. Viral titers in the supernatants and viral protein expression in the cells were examined at the indicated time points by TCID_50_ assays (**A**) and western blot (**B**), respectively. (**C**,**D**) Plaque morphology of recombinant JEV. BHK-21 cell monolayers were infected with the indicated viruses for an analysis of plaque morphology. The plaques were stained with crystal violet at 5 dpi (**C**) and the plaque diameters were measured and plotted (**D**). The significant differences between the groups were tested by Student’s *t*-test.

To examine the effects of an HA-tag insertion and NS1’ deletion on viral replication, the difference in the replication kinetics between JEV-HA/NS2A/∆NS1’ and JEV-WT was determined in BHK-21 cells. The supernatants from JEV-infected cells were collected at 24, 48, 72, and 96 hpi, and subjected to titration for viral replication. Both viruses displayed similar growth curves. The viral titers of JEV-HA/NS2A/∆NS1’ were relatively lower than those of JEV-WT; however, there were no significant differences between the two viruses (Figure 3A). The abundance of NS5 expression was examined by western blotting and no significant differences were observed between JEV-HA/NS2A/∆NS1’ and JEV-WT (Figure 3B). HA-NS2A expression was detectable in the cells infected with JEV-HA/NS2A/∆NS1’, but not with JEV-WT (Figure 3B), confirming that NS2A was labeled with HA-tag. To further analyze the replication characteristics, plaque morphology was compared between JEV-HA/NS2A/∆NS1’ and JEV-WT. The size of plaque formed by JEV-HA/NS2A/∆NS1’ was 2.05 ± 0.03 mm, which was relatively smaller than that (2.11 ± 0.03 mm) of JEV-WT, but there were no significant differences between the two viruses (Figure 3C,D). Furthermore, the difference in the replication kinetics and plaque morphology between JEV-HA/NS2A and JEV-WT was determined in BHK-21 cells and no significant differences were observed between the two viruses (Supplementary Figure S2). Overall, these data indicate that the HA-tag insertion and NS1’ deletion showed no significant effects on viral replication, thereby suggesting that JEV-HA/NS2A/∆NS1’ might be used as a model for an analysis of the biological characteristics and functions of NS2A in vitro during viral infection.

### 3.4. Detection of HA-NS2A Localization to the ER

**Figure 4:**
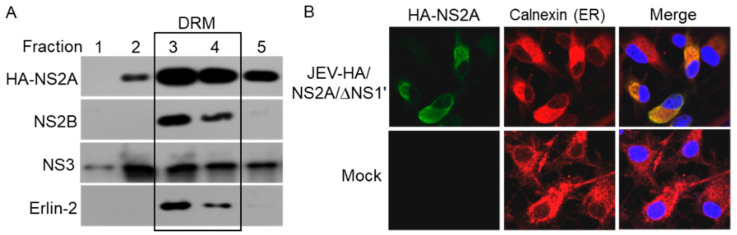
HA-NS2A localization to the ER. (**A**) BHK-21 cells were infected with JEV-HA/NS2A/∆NS1’ and harvested at 48 hpi for an isolation of the detergent-resistant membranes (DRM). The presence of the indicated proteins in the DRM was detected by western blots. (**B**) BHK-21 cells were infected with JEV-HA/NS2A/∆NS1’ and subjected to an immunofluorescence assay at 24 hpi. HA-NS2A (green) and the ER (red) were detected with anti-HA and anti-calnexin antibodies, respectively. The nuclei (blue) were stained with DAPI.

Flavivirus NS2A is a membrane protein localized in the ER with five membrane span regions [8,27]. Therefore, we used a model of JEV-HA/NS2A/∆NS1’ to determine whether JEV NS2A was able to localize to the ER. To this end, BHK-21 cells were infected with JEV-HA/NS2A/∆NS1’ and subjected to detergent-resistant membrane isolation. The detergent-resistant fractions corresponding to the DRM within the ER [28] were collected from the top gradient (the lowest density) of the sucrose density gradient tube and subjected to western blot analysis of the presence of HA-NS2A by anti-HA antibodies (Figure 4A). In addition, the DRM was identified by Erlin-2-specific antibodies—an ER marker [28]. A portion of HA-NS2A was detected in fractions 3 and 4 where Erlin-2 was present, suggesting that HA-NS2A could localize to the ER, in agreement with the ER association of flavivirus NS2A [8,27]. It has been established that flavivirus NS2B and NS3 are localized to the ER [23,29]. The localization of JEV NS2B and NS3 was also examined to the DRM and NS2B and a partial of NS3 was found to be present in the fractions 3 and 4, suggesting that JEV NS2B and NS3 were localized to the ER. To further confirm ER localization of HA-NS2A, BHK-21 cells were infected with JEV-HA/NS2A/∆NS1’ and subjected to an analysis of the subcellular distribution of HA-NS2A by IFA with anti-HA antibodies. Calnexin was used as an ER marker [8]. In addition, the IFA results showed that HA-NS2A was mainly distributed around the nucleus in the cytoplasm and colocalized with calnexin (Figure 4B). Overall, these results demonstrated that JEV NS2A was able to localize to the ER, indicating that JEV-HA/NS2A/∆NS1’ could be used as a model to study the biological characteristics of NS2A in vitro during viral infection.

### 3.5. Detection of the Interaction between HA-NS2A and Viral Proteins 

**Figure 5:**
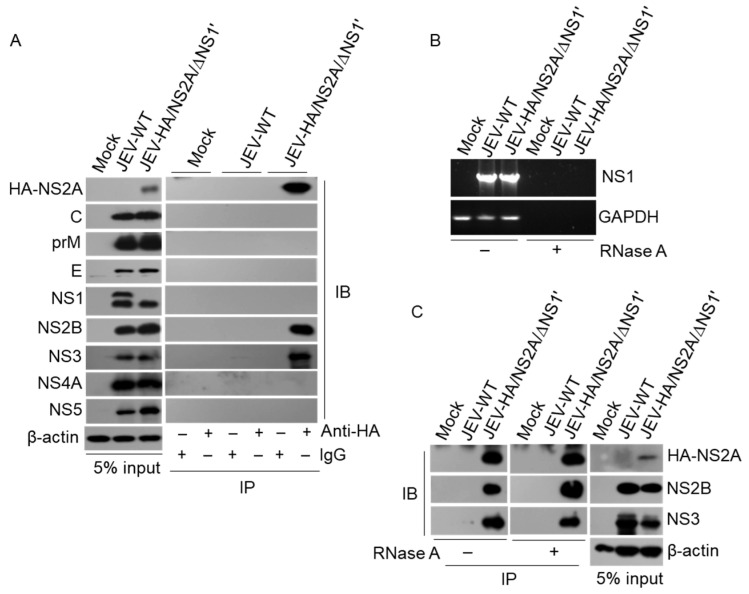
Interaction of HA-NS2A with viral proteins. BHK-21 cells were infected with JEV and harvested at 48 hpi for a co-immunoprecipitation assay. (**A**) The cell lysates were incubated with anti-HA antibodies and the immunoprecipitated proteins were detected with the indicated antibodies against different viral proteins. (**B**) The cell lysates were pre-treated with RNase A and the presence of NS1 and GAPDH RNAs was examined by RT-PCR. (**C**) The cells lysates were pre-treated with RNase A and subsequently subjected to co-immunoprecipitation with anti-HA antibodies. The immunoprecipitated proteins were detected by the indicated antibodies against different viral proteins.

Flavivirus NS2A interacts with viral proteins for replication [27]. Therefore, we examined the interaction of JEV NS2A with viral proteins by co-immunoprecipitation using anti-HA antibodies. To this end, BHK-21 cells were infected with JEV-HA/NS2A/∆NS1’ and subjected to a co-immunoprecipitation analysis. The immunoprecipitated proteins were detected by antibodies against different viral proteins. As shown in Figure 5A, NS2B and NS3, but not other viral proteins, were pulled down with HA-NS2A by anti-HA antibodies. It has been established that flavivirus NS2A can bind to and recruit viral RNA to the viral replication site [7,8]. To explore whether the interaction between HA-NS2A with NS2B and NS3 was mediated by viral RNA, the cell lysates were pre-treated with RNase A to degrade RNAs and subsequently subjected to a co-immunoprecipitation analysis. A RT-PCR analysis of viral NS1 RNA showed that no RNA was detectable in the cell lysates pre-treated with RNase A (Figure 5B). This finding suggests that all RNAs were completely degraded, including the RNA of glyceraldehyde 3-phosphate dehydrogenase (GAPDH). An analysis of the proteins immunoprecipitated from the RNase A-treated cell lysates indicated that NS2B and NS3 remained pulled down with HA-NS2A by anti-HA antibodies (Figure 5C). Overall, these data suggest that NS2A could directly interact with NS2B and NS3, suggesting that JEV-HA/NS2A/∆NS1’ could be used as a model to study NS2A functionality in vitro during viral infection.

## 4. Discussion

One major challenge associated with studying the functions of flavivirus NS2A that contributes to viral replication and pathogenesis is a lack of NS2A-specific antibodies. One previous study described that the authors failed to develop antibodies for ZIKV NS2A after multiple attempts using various approaches [8]. We also experienced difficulty in generating JEV NS2A-specific antibodies. In this study, we constructed recombinant JEV-HA/NS2A/∆NS1’ with HA-tagged NS2A to overcome this challenge.

HA-tag was fused to the N-terminus of NS2A at the intergenic junction between NS1 and NS2A to construct the recombinant JEV-HA/NS2A/∆NS1’. Since both NS1 and NS2A play important roles in the viral life cycle, the correct cleavage between NS1 and NS2A is important to their biological functionality. To ensure the correct cleavage between the C-terminus of NS1 and the N-terminus of HA-NS2A, the first three amino acids of “FNG” from the N-terminus of NS2A was inserted as a linker, into the junction site between NS1 and HA-NS2A. A western blot analysis of NS1 expression demonstrated that the molecular weight of NS1 of JEV-HA/NS2A/∆NS1’ was identical to that of JEV-WT, suggesting that the correct cleavage occurred between NS1 and HA-NS2A. In addition, the HA-tag insertion did not affect the cleavage between NS1 and HA-NS2A. 

It has been established that an RNA pseudoknot-mediated ribosomal frameshift event occurs between codons 8 and 9 of the NS2A gene, resulting in synthesis of the derivative NS1’ [17,18]. The HA-tag insertion not only generated HA-NS2A of interest, but also conferred the unwanted production of HA-NS1’, which can interfere with the results when anti-HA antibodies were used to analyze the biological characteristics and functions of HA-NS2A. To silence HA-NS1’ production, A30P substitution was introduced at NS2A residue 30, which disrupted the frameshift-stimulating pseudoknot structure, and abolished the synthesis of HA-NS1’ [18]. 

Stability of the HA-tag insertion and A30P substitution in JEV-HA/NS2A/∆NS1’ was important for the analysis of NS2A functions. DNA sequencing demonstrated that the HA-tag insertion and A30P substitution were present after six passages, showing good stability of JEV-HA/NS2A/∆NS1’. Analysis of the replication kinetics and plaque morphology indicated that JEV-HA/NS2A/∆NS1’ exhibited growth properties and a plaque morphology similar to JEV-WT in BHK-21 cells, demonstrating that the HA-tag insertion and A30P substitution had no significant effect on viral replication. In addition, the elimination of NS1’ expression by A30P substitution did not display any significant effects on viral replication. This finding is in agreement with a previous observation that ablating NS1’ expression does not affect viral replication properties and plaque morphology in mammalian and mosquito cells [20]. Based on these observations, it was determined that JEV-HA/NS2A/∆NS1’ could be used as a model for analyzing the biological characteristics and functions of NS2A in vitro during JEV infection. 

We first examined whether JEV NS2A could localize to the ER using the JEV-HA/NS2A/∆NS1’ model. Detergent-resistant membrane isolation demonstrated that a portion of HA-NS2A was distributed to the DRM, suggesting that HA-NS2A could localize to the ER, in agreement with the ER association of flavivirus NS2A [8,27]. Moreover, flavivirus replication and virion assembly occur at the ER. The membrane association of viral nonstructural proteins, including NS2A, NS1, NS2B, NS4A, and NS4B, which reside on the ER membrane, are thought to be important for structural remodeling of the ER to establish the viral replication organelles that are structurally required for viral replication [27]. A peptide (Dens25) in the N-terminus of DENV2 NS2A possesses membrane insertion properties in vitro and is likely involved in membrane modulation [11,30]; however, whether flavivirus NS2A plays a role in establishing the organelle associated with viral replication remains unclear [27]. The JEV-HA/NS2A/∆NS1’ model generated in this study could be useful for exploring the role of flavivirus NS2A in establishing the viral replication organelles. 

Flavivirus NS2A interacts with viral proteins for replication. The NS2A of DENV, ZIKV, and YFV has been documented to participate in virion assembly [6,7,8,9,10,11,12,13]. Recent studies describe that DENV and ZIKV NS2A recruits viral RNA, structural proteins, and protease (NS2B-NS3) to the site of virion assembly and coordinates nucleocapsid and virus formation [7,8]. The interaction between JEV NS2A and viral proteins is unknown. In taking advantage of the JEV-HA/NS2A/∆NS1’ model, we analyzed the interaction between NS2A and viral proteins during JEV infection through co-immunoprecipitation with anti-HA antibodies. NS2B and NS3 were pulled down by HA-NS2A, preliminarily suggesting that NS2A could interact with NS2B and NS3. However, we did not observe the interaction of NS2A with prM and E, which has been observed in ZIKV-infected cells [8]. Whether JEV NS2A interacts with prM and E remains to be determined in the future. JEV NS2A is involved in viral growth, tissue tropism, virulence, and pathogenesis [19,20,31]. However, the underlying mechanisms are poorly understood. The model of JEV-HA/NS2A/∆NS1’ generated in this study may be useful for exploring the roles of NS2A in JEV replication and pathogenesis. 

Although we demonstrated that JEV-HA/NS2A/∆NS1’ can serve as a model to analyze the biological characteristics and functions of NS2A in vitro during JEV infection, somewhat shortness was present in this study. The replication of JEV-HA/NS2A/∆NS1’ was examined only in BHK-21 cells, but not in other cell types, such as cells derived from JEV natural hosts, as well as not in animal model. Whether the replication of JEV-HA/NS2A/∆NS1’ in BHK-21 cells was identical to those in other cell types and animal model remained unclear. In addition, it is known that NS1’ plays important role in JEV pathogenicity and host antiviral response [20,32]. For example, NS1’ interacts with host CDK1 protein to dampen the cellular antiviral response [33]. The elimination of NS1’ production might consequently generate a phenotype that could interfere with the biological characteristics and functions of NS2A observed from JEV-HA/NS2A/∆NS1’ model. Therefore, this limitation of JEV-HA/NS2A/∆NS1’ model should be kept in mind when this model is used for analyzing the biological characteristics and functions of NS2A. In conclusion, recombinant JEV-HA/NS2A/∆NS1’ was constructed by fusing the HA-tag to the N-terminus of NS2A at the intergenic junction between NS1 and NS2A to overcome a lack of NS2A-specific antibodies. An A30P substitution was introduced at residue 30 of NS2A to abolish synthesis of the unwanted HA-NS1’. The insertion of the HA-tag and A30P substitution were stably present in JEV-HA/NS2A/∆NS1’ after six passages and showed no significant effects on viral replication or plaque morphology. HA-NS2A was able to localize to the ER and interact with viral NS2B and NS3 during JEV infection. These data suggest that JEV-HA/NS2A/∆NS1’ can serve as a model to analyze the biological characteristics and functions of NS2A in vitro during JEV infection.

## Data Availability

Not applicable.

## Supplementary Materials

The following supporting information can be downloaded at: https://www.mdpi.com/article/10.3390/v14040706/s1, Table S1: Primer sequences; Figure S1: Schematic diagram of construction of recombinant JEV; Figure S2: Replication of JEV-WT and JEV-HA/NS2A in BHK-21 cells.

## References

[1] Mackenzie J.S., Gubler D.J., Petersen L.R. (2004). Emerging flaviviruses: The spread and resurgence of Japanese encephalitis, West Nile and dengue viruses. Nat. Med..

[2] Nga P.T., Parquet M.d.C., Cuong V.D., Ma S.-P., Hasebe F., Inoue S., Makino Y., Takagi M., Nam V.S., Morita K. (2004). Shift in Japanese encephalitis virus (JEV) genotype circulating in northern Vietnam: Implications for frequent introductions of JEV from Southeast Asia to East Asia. J. Gen. Virol..

[3] Satchidanandam V. (2020). Japanese Encephalitis Vaccines. Curr. Treat. Options Infect. Dis..

[4] van den Hurk A.F., Ritchie S.A., Mackenzie J.S. (2009). Ecology and geographical expansion of Japanese encephalitis virus. Annu. Rev. Entomol..

[5] Conde J.N., Silva E.M., Barbosa A.S., Mohana-Borges R. (2017). The Complement System in Flavivirus Infections. Front. Microbiol..

[6] Xie X., Gayen S., Kang C., Yuan Z., Shi P.Y. (2013). Membrane topology and function of dengue virus NS2A protein. J. Virol..

[7] Xie X., Zou J., Zhang X., Zhou Y., Routh A.L., Kang C., Popov V.L., Chen X., Wang Q.Y., Dong H. (2019). Dengue NS2A Protein Orchestrates Virus Assembly. Cell Host Microbe.

[8] Zhang X., Xie X., Xia H., Zou J., Huang L., Popov V.L., Chen X., Shi P.-Y. (2019). Zika Virus NS2A-Mediated Virion Assembly. mBio.

[9] Leung J.Y., Pijlman G.P., Kondratieva N., Hyde J., Mackenzie J.M., Khromykh A.A. (2008). Role of nonstructural protein NS2A in flavivirus assembly. J. Virol..

[10] Mackenzie J.M., Khromykh A.A., Jones M.K., Westaway E.G. (1998). Subcellular localization and some biochemical properties of the flavivirus Kunjin nonstructural proteins NS2A and NS4A. Virology.

[11] Nemésio H., Villalaín J. (2014). Membrane interacting regions of Dengue virus NS2A protein. J. Phys. Chem. B.

[12] Voßmann S., Wieseler J., Kerber R., Kümmerer B.M. (2015). A basic cluster in the N terminus of yellow fever virus NS2A contributes to infectious particle production. J. Virol..

[13] Wu R.H., Tsai M.H., Chao D.Y., Yueh A. (2015). Scanning mutagenesis studies reveal a potential intramolecular interaction within the C-terminal half of dengue virus NS2A involved in viral RNA replication and virus assembly and secretion. J. Virol..

[14] Muñoz-Jordan J.L., Sánchez-Burgos G.G., Laurent-Rolle M., García-Sastre A. (2003). Inhibition of interferon signaling by dengue virus. Proc. Natl. Acad. Sci. USA.

[15] Xia H., Luo H., Shan C., Muruato A.E., Nunes B.T.D., Medeiros D.B.A., Zou J., Xie X., Giraldo M.I., Vasconcelos P.F.C. (2018). An evolutionary NS1 mutation enhances Zika virus evasion of host interferon induction. Nat. Commun..

[16] Yoon K.J., Song G., Qian X., Pan J., Xu D., Rho H.S., Kim N.S., Habela C., Zheng L., Jacob F. (2017). Zika-Virus-Encoded NS2A Disrupts Mammalian Cortical Neurogenesis by Degrading Adherens Junction Proteins. Cell Stem Cell.

[17] Firth A.E., Atkins J.F. (2009). A conserved predicted pseudoknot in the NS2A-encoding sequence of West Nile and Japanese encephalitis flaviviruses suggests NS1’ may derive from ribosomal frameshifting. Virol. J..

[18] Melian E.B., Hinzman E., Nagasaki T., Firth A.E., Wills N.M., Nouwens A.S., Blitvich B.J., Leung J., Funk A., Atkins J.F. (2010). NS1’ of flaviviruses in the Japanese encephalitis virus serogroup is a product of ribosomal frameshifting and plays a role in viral neuroinvasiveness. J. Virol..

[19] Tajima S., Taniguchi S., Nakayama E., Maeki T., Inagaki T., Lim C.K., Saijo M. (2020). Amino Acid at Position 166 of NS2A in Japanese Encephalitis Virus (JEV) is Associated with In Vitro Growth Characteristics of JEV. Viruses.

[20] Ye Q., Li X.F., Zhao H., Li S.H., Deng Y.Q., Cao R.Y., Song K.Y., Wang H.J., Hua R.H., Yu Y.X. (2012). A single nucleotide mutation in NS2A of Japanese encephalitis-live vaccine virus (SA14-14-2) ablates NS1’ formation and contributes to attenuation. J. Gen. Virol..

[21] Tu Y.C., Yu C.Y., Liang J.J., Lin E., Liao C.L., Lin Y.L. (2012). Blocking double-stranded RNA-activated protein kinase PKR by Japanese encephalitis virus nonstructural protein 2A. J. Virol..

[22] Fan W., Wu M., Qian S., Zhou Y., Chen H., Li X., Qian P. (2016). TRIM52 inhibits Japanese Encephalitis Virus replication by degrading the viral NS2A. Sci. Rep..

[23] Xing H., Xu S., Jia F., Yang Y., Xu C., Qin C., Shi L. (2020). Zika NS2B is a crucial factor recruiting NS3 to the ER and activating its protease activity. Virus Res..

[24] Zhang X., Xie X., Zou J., Xia H., Shan C., Chen X., Shi P.Y. (2019). Genetic and biochemical characterizations of Zika virus NS2A protein. Emerg. Microbes Infect..

[25] Zhu Z., Shi Z., Yan W., Wei J., Shao D., Deng X., Wang S., Li B., Tong G., Ma Z. (2013). Nonstructural protein 1 of influenza A virus interacts with human guanylate-binding protein 1 to antagonize antiviral activity. PLoS ONE.

[26] Bakhache W., Neyret A., Bernard E., Merits A., Briant L. (2020). Palmitoylated Cysteines in Chikungunya Virus nsP1 Are Critical for Targeting to Cholesterol-Rich Plasma Membrane Microdomains with Functional Consequences for Viral Genome Replication. J. Virol..

[27] Ci Y., Shi L. (2021). Compartmentalized replication organelle of flavivirus at the ER and the factors involved. Cell. Mol. Life Sci. CMLS.

[28] Wu M.J., Shanmugam S., Welsch C., Yi M. (2019). Palmitoylation of Hepatitis C Virus NS2 Regulates Its Subcellular Localization and NS2-NS3 Autocleavage. J. Virol..

[29] Li Y., Li Q., Wong Y.L., Liew L.S., Kang C. (2015). Membrane topology of NS2B of dengue virus revealed by NMR spectroscopy. Biochim. Biophys. Acta.

[30] Fajardo-Sánchez E., Galiano V., Villalaín J. (2017). Spontaneous membrane insertion of a dengue virus NS2A peptide. Arch. Biochem. Biophys..

[31] Takamatsu Y., Morita K., Hayasaka D. (2015). A Single Amino Acid Substitution in the NS2A Protein of Japanese Encephalitis Virus Affects Virus Propagation In Vitro but Not In Vivo. J. Virol..

[32] Zhou D., Li Q., Jia F., Zhang L., Wan S., Li Y., Song Y., Chen H., Cao S., Ye J. (2020). The Japanese Encephalitis Virus NS1’ Protein Inhibits Type I IFN Production by Targeting MAVS. J. Immunol..

[33] Li Q., Zhou D., Jia F., Zhang L., Ashraf U., Li Y., Duan H., Song Y., Chen H., Cao S. (2021). Japanese Encephalitis Virus NS1’ Protein Interacts with Host CDK1 Protein to Regulate Antiviral Response. Microbiol. Spectr..

