# Supplementary material for: Construction of a Recombinant Japanese Encephalitis Virus with a Hemagglutinin-Tagged NS2A: A Model for an Analysis of Biological Characteristics and Functions of NS2A during Viral Infection
Source: Viruses. 2022 Mar 29;14(4):706. doi: 10.3390/v14040706 (PMC9024733; doi:10.3390/v14040706)
Supplement: Supplementary file 1 [file viruses-14-00706-s001.zip › Supplementary Figure S2's figure caption.pdf]

Supplementary Figure S2. Replication of JEV-WT and JEV-HA/NS2A in BHK-21 cells. (A) BHK-21 cells were infected with the indicated viruses at an MOI of 0.01. Viral titers in the supernatants were examined at the indicated time points by $TCID_{50}$ assays. (B) Plaque morphology of recombinant JEV. BHK-21 cell monolayers were infected with the indicated viruses for an analysis of plaque morphology. The plaques were stained with crystal violet at 5 dpi.
