# Supplementary material for: Construction of a Recombinant Japanese Encephalitis Virus with a Hemagglutinin-Tagged NS2A: A Model for an Analysis of Biological Characteristics and Functions of NS2A during Viral Infection
Source: Viruses. 2022 Mar 29;14(4):706. doi: 10.3390/v14040706 (PMC9024733; doi:10.3390/v14040706)
Supplement: Supplementary file 1 [file viruses-14-00706-s001.zip › Supplementary Table S1 Primer sequences.pdf]

Supplementary Table S1 Primer sequences

Primer name	Primer sequence (5’-3’)	Purpose
HA-NS2A-F	TACGATGTTCCAGATTACGCTGGTGGCGGATTCAACGGCGAAATGATTGACCCTTTTCAG	Primers for amplification of HA-NS2A
HA-NS2A-R	ATCTGGAACATCGTATGGGTAGCCGTTGAAAGCGTCAACCTGTGACCTAACGAGTGTTGT	Primers for amplification of HA-NS2A
HA-A30P-F	CCAAGATTGACGATTCCTGCGGTTTTGGGGGCTCTAC	Primers for amplification of HA-A30P
HA-A30P-R	CAGGAATCGTCAATCTTGGCGTCCACCTCTTGCGAAG	Primers for amplification of HA-A30P
JEV-I-F	GCGCCTAATACGACTCACTATAGGGAGAAGTTTATCTGTGTGAACTTCTTG	Primers for amplification of fragment I
JEV-I-R	CCTTGTGTGATCCAAGACATTCCCCCAAAGA	Primers for amplification of fragment I
JEV-II-F	TCTTTGGGGGAATGTCTTGGATCACACAAGG	Primers for amplification of fragment II
JEV-II-R	CCAGACCTTCCATGGAACACCGGGATCATCAATCAAGTGAAA	Primers for amplification of fragment II
JEV-III-F	TTTCACTTGATTGATGATCCCGGTGTTCCATGGAAGGTCTGG	Primers for amplification of fragment III
JEV-III-R	AAGGAGGGCTTGTCAGCGTTCTTGATGAGAGTCCA	Primers for amplification of fragment III
JEV-IV-F	TGGACTCTCATCAAGAACGCTGACAAGCCCTCCTT	Primers for amplification of fragment IV
JEV-IV-R	AGATCCTGTGTTCTTCCTCACCACCAGCTACATACT	Primers for amplification of fragment IV
