# Supplementary figures and images for: Construction of a Recombinant Japanese Encephalitis Virus with a Hemagglutinin-Tagged NS2A: A Model for an Analysis of Biological Characteristics and Functions of NS2A during Viral Infection
Source: Viruses. 2022 Mar 29;14(4):706. doi: 10.3390/v14040706 (PMC9024733; doi:10.3390/v14040706)

Supplementary Fig. S1 Schematic diagram of construction of recombinant JEV

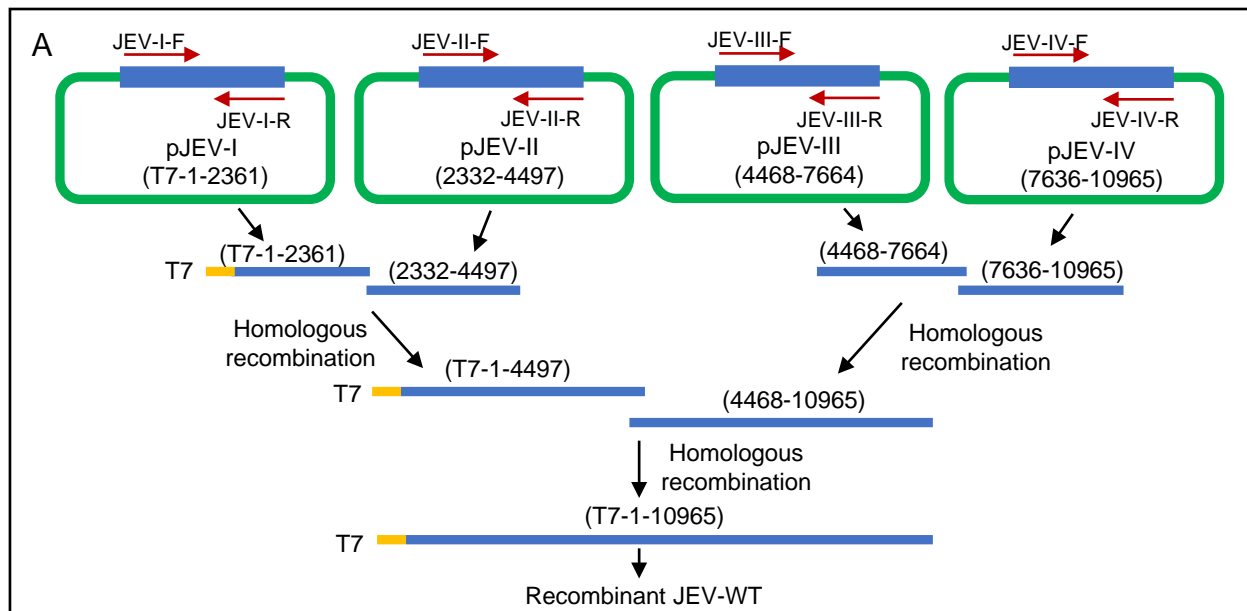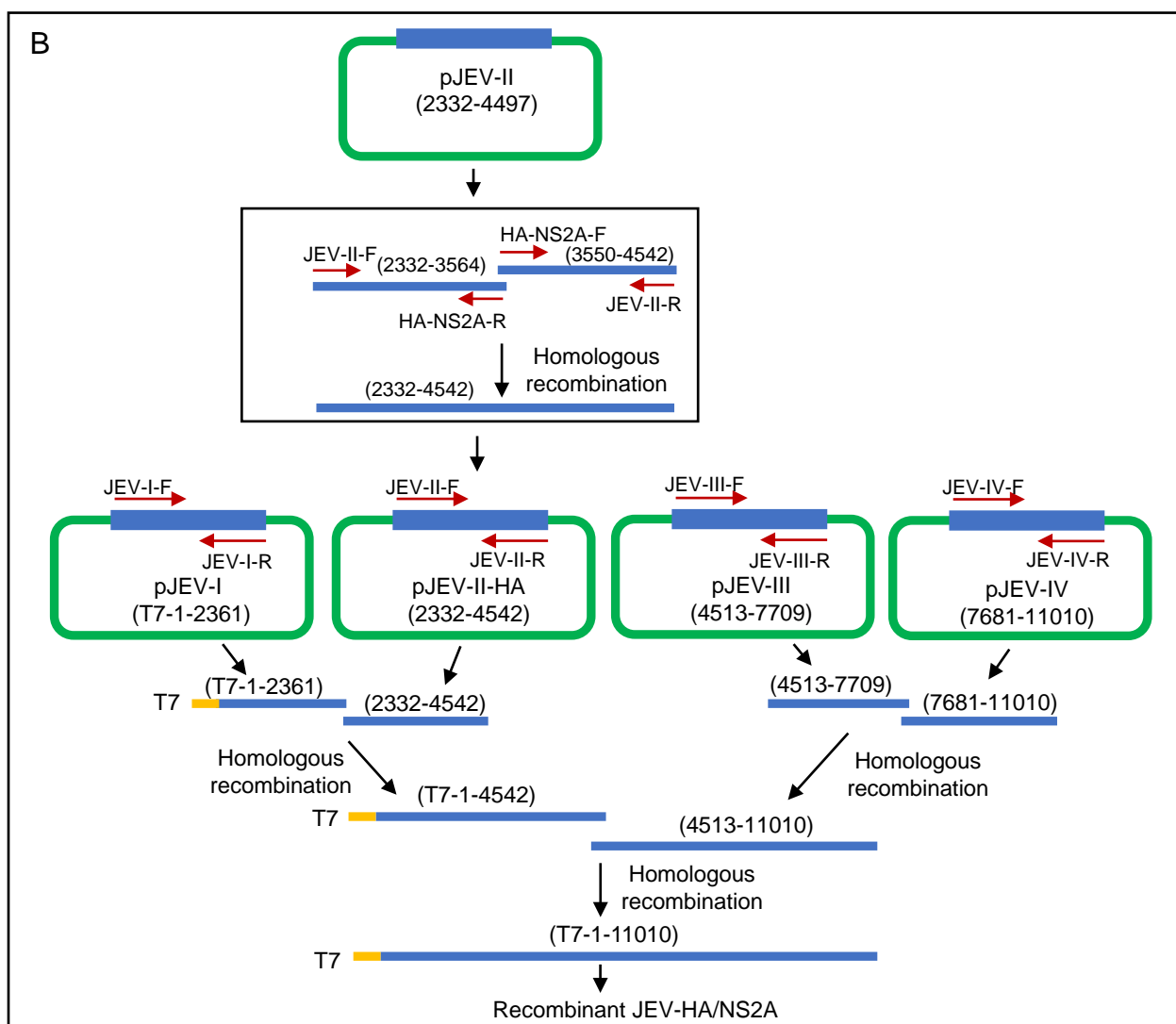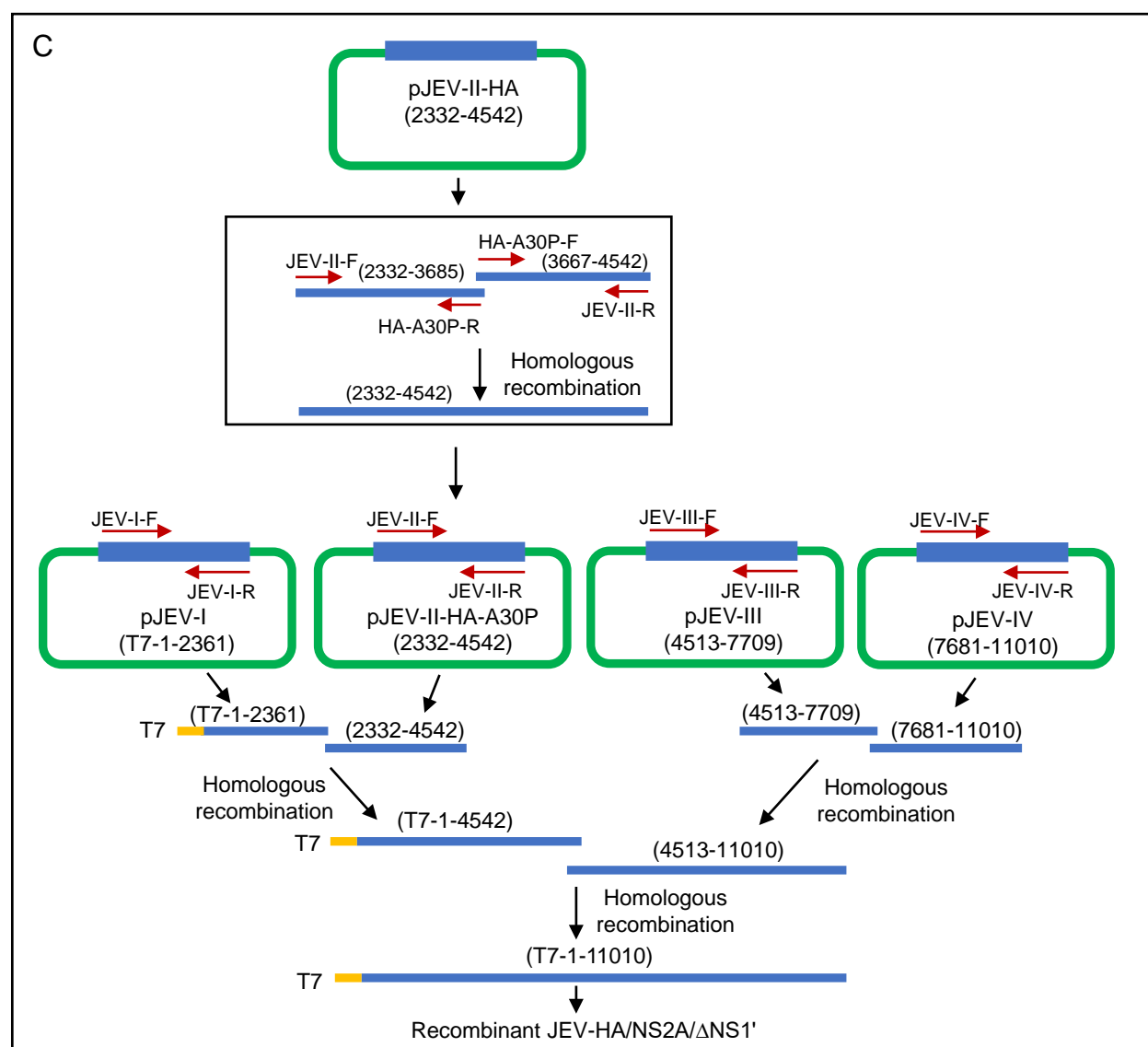

Supplement: Supplementary file 1 [file viruses-14-00706-s001.zip › Supplementary Fig. S1.pdf]

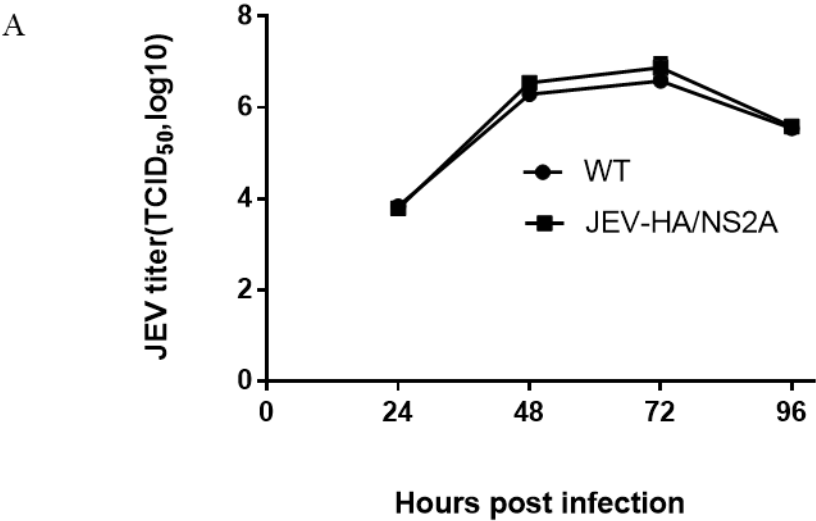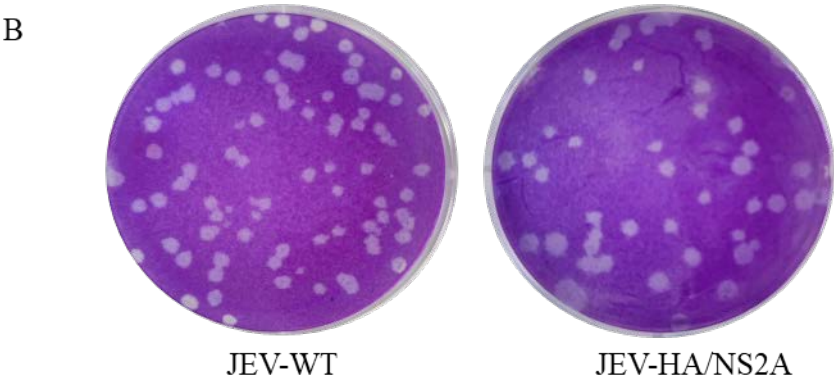

Supplement: Supplementary file 1 [file viruses-14-00706-s001.zip › Supplementary Fig. S2.pdf]
